# Role of basophil activation test for monitoring the immunological changes during desnsitization to cow’s milk: a case report

**DOI:** 10.1186/2045-7022-1-S1-P95

**Published:** 2011-08-12

**Authors:** Valentina Pecora, Eleonora Nucera, Amira Colagiovanni, Alessandro Buonomo, Tiziana De Pasquale, Sonia Musumeci, Angela Rizzi, Arianna Aruanno, Lucilla Pascolini, Anna Giulia Ricci, Vito Sabato, Domenico Schiavino

**Affiliations:** 1 Policlinico A. Gemelli, Allergy Department, Rome, Italy

## 

The basophil activation test (BAT) has proven to be a useful tool for the diagnosis of IgE-mediated food allergy and the evaluation of clinical tolerance in food allergic patients. Until now, successful oral desensitization to food allergen has been correlated with changes in cytokine production (IL-4 and INF-γ) and allergen-specific IgE and IgG4 antibodies.

We report a case of a 12-year-old male affected by cow’s milk allergy. The diagnosis was based on a positive allergological work-up, included skin prick test with commercial food extracts (Alk-Abellò) and fresh cow’s milk (prick-by-prick method), detection in serum of total and specific IgE (ImmunoCAP System, Phadia), basophil activation test and finally a double-blind placebo-controlled food challenge. Skin prick test, specific IgE, BAT and oral food challenge were positive and so we decided to carry on a sublingual-oral desensitization treatment with cow’s milk, performed according to standardized protocol. In order to evaluate the immunological changes occurred during the immunotherapy, we performed further laboratory tests, included the detection in serum of total and specific IgE and BAT at the end of protocol.

Desensitization was successfully carried out within 12 months, reaching the maximum dose of 150 ml of cow’s milk without side effects. The patient showed a decrease of specific IgE levels without reaching normal values and an increase of specific IgG4 levels; whereas the BAT starting from a positive value of 59% became negative after 12 months of treatment.

This case report shows how it is possible to monitor allergen-specific basophil responses using the flow cytometry in order to identify an acquired tolerance induced by desensitization. Although further studies are needed, it was interesting to note that the basophil activation test seems to be more sensitive and characterized by a close correlation with the clinical tolerance.

